# User-Focused Monitoring as a Strategy for Involvement and Mental Health Service Development: An Analysis of Swedish Monitoring Reports

**DOI:** 10.1007/s40737-022-00268-6

**Published:** 2022-03-23

**Authors:** Hilda Näslund, Katarina Grim, Urban Markström

**Affiliations:** 1 Department of Social Work, Umeå University, Umeå, Sweden; 2 Department of Social and Psychological Studies, Karlstad University, Karlstad, Sweden

**Keywords:** Mental health, User involvement, User-focused monitoring, Service evaluation

## Abstract

User-focused monitoring (UFM) is a method of evaluating mental health services, conducted by people with lived experience of mental ill health. Research on UFM and on user involvement focused on service monitoring and evaluation is lacking. This study addresses this knowledge gap by examining UFM as a strategy for user involvement. More specifically, this study aims to synthesize patterns in UFM reports to characterize the phenomenon, as well as to further discuss negotiation processes and political opportunities in UFM. The empirical material consists of 136 Swedish UFM reports that have been analyzed in two steps: All reports were mapped according to general characteristics and a sample of 20 reports were selected to provide additional information on the method. This study has been conducted in collaboration with actors representing the user movement and municipality-based mental health services. Our analysis shows that long-term contracts between user organizations and service providers are important to create a sustainable implementation of UFM. However, strategies to protect user autonomy must be carefully considered and employed in relation to such collaborations. We further highlight the risks of a restricted focus on consumer satisfaction, and discuss the current development towards including follow-ups in the UFM process as a strategy for counteracting tokenism.

## Introduction

Today, there is broad acceptance of the need to include service user perspectives as an essential component in quality development of mental health services [1–3]. A number of practices have been established to strengthen user involvement, including so-called user-focused monitoring (UFM), which is a method of evaluating services, conducted by people with lived experience of mental ill health [4, 5]. UFM presents an interesting case for studying user involvement as it is carried out at the operational level of the service organization and thus enables an analysis of how service users' experiences can be integrated in development work. Interest in UFM has increased in Swedish mental health services in the past decade. Still, studies on the phenomenon are lacking. In this study, we analyze UFM reports produced in Sweden, to characterize the phenomenon. This study is carried out in collaboration with actors from the user movement and municipality-based mental health services.

The World Health Organization (WHO) promotes the engagement of service users in the implementation of policy at the national level and in the monitoring of services at the local level [6]. In Sweden, user involvement is presented as a priority issue in government commissions of inquiry [7, 8]. The experiences and preferences of service users are further necessary in order to develop the integrated knowledge base that underlies evidence-based practice [9–11]. A range of user involvement initiatives have been developed in the mental health sector, ranging from those enacted at the individual level, to those enacted at the systemic level ([12], p. 212). In Sweden, the establishment of user councils, projects focused on hiring peer supporters in services, and initiatives linked to person-centered care and shared decision-making exemplify practices developed to strengthen user involvement [13–15]. Meanwhile, knowledge of methods that seek to influence the quality of services from a service user perspective is limited [16–18]. In particular, research on user involvement at an organizational level [16] and research on service monitoring and evaluation [17] are lacking.

### User-Focused Monitoring

UFM is described as a systematic and independent method of reviewing care and support services, conducted by people with lived experience of mental ill health [4, 5]. UFM was originally developed in England during the 1990’s [4] and variations of the practice were later translated to the Swedish context. Typically, UFM is commissioned by a mental health service provider who invite user monitors to assess the service as part of their development work. UFM results and proposals are thereby meant to be integrated in development efforts, by contributing to the inclusion of a service user perspective. There are examples of UFM projects carried out by public sector actors or by a research and development unit, but the majority of UFMs in Sweden are conducted by large user organizations in more populated areas with a well-established user movement. User organizations recruit user monitors through publicly or internally posted information, and interview potential candidates for the role. The user organizations further provide training for the user monitors. This training is typically around two days long and is focused on the intent of the method, basic research and evaluation methods and practicalities connected to the UFM process. Teams of user monitors are contracted to conduct formal evaluations and produce a report on a specific service context or program from a service user perspective. Within community mental health, services such as day centers, vocational programs, group homes, and housing support are often evaluated. UFM is also conducted within region-based psychiatry such as inpatient and outpatient care. User monitors decide on the focus of the UFM and carry out interviews or surveys targeting service users within the given service context. Specifics of the method may vary, but the primary idea is that service users should lead the process at every stage, through designing and conducting the UFM, as well as analyzing and presenting the results with associated recommendations [4, 19].

UFM practices draw on research methodology and thus have similarities to user involvement in research, which is a prolific field of study (e.g. [20–23]). However, research on UFM is lacking even though the need to evaluate such methods has been discussed [6]. This study examines UFM as a strategy for user involvement. More specifically, the study aims to synthesize patterns in UFM reports to characterize the phenomenon and to further discuss negotiation processes and political opportunities in UFM.

### The Swedish Context

During the past few decades, interest in UFM has steadily increased in Sweden and around 30 UFM reports are now conducted yearly in the mental health sector. The welfare system in Sweden has been discussed as a social democratic regime [24], with the public sector accounting for most welfare interventions. Even though the Swedish welfare system is today developing towards an increase of private and non-profit providers, the public sector remains dominant in mental health service provisioning [25]. The Swedish welfare system is highly sectorized, with two main mental health service providers. Municipal community mental health services offer residential and vocational support, and region-based psychiatric services offer in- and outpatient care. Close relationships between public authorities and the user movement further mark the context for UFM in Sweden [26].

Structures for user involvement have been established in the Swedish mental health service system at different levels. Commonly the main providers of UFM, Swedish mental health user organizations have, since the 1990s, been engaged in government projects and commissions of inquiry related to mental health [25]. During past decades, a growing number of user organizations have been established in Sweden, many of which are diagnosis-specific [26]. There are examples of mental health non-profit organizations, such as Fountain House, that are not user-led [25]. However, the user movement in Sweden is based on user-led membership organizations, connected to the domestic popular mass movement tradition [26]. Today, these organizations have a key role in quality development and democratization of the welfare system. UFM relates to both these dimensions. The method aims to improve the quality of services by including the preferences and experiences of the end-user in development processes. User involvement methods can also contribute to increasing the influence of user groups and provide user organizations transparency into welfare services. Moreover, the welfare sector’s governance structure has undergone change, where a hierarchical tradition has been replaced by more network-based governance, where a part of state responsibilities is delegated to other actors [27]. UFM connects to these patterns, as actors representing the user movement assume a consultative role in processes of developing welfare services. Such changes require user organizations to simultaneously assume roles as partners and critical monitors of welfare services (cf. [28]).

UFM further reflects a general trend to where perspectives central to user movements such as recovery, empowerment, and experiential knowledge have received increasing attention in the Swedish mental health service system [29, 30]. The increased status of experiential knowledge could enable the inclusion of alternative perspectives in the development of mental health services, based on the user collectives’ knowledge. This pattern further legitimizes and contributes to demand for UFM, since the practice is grounded in service users’ experiential knowledge.

### Seizing Political Opportunities Through Negotiations

The strategies for influence chosen by user collectives and the outcomes of their efforts relate to the political opportunities that are available in an institutional environment. In political opportunity theory, power relations and political processes that enable political action are at the core. Social movement actions and repertoires are seen as performed in political contexts that are shaped by political opportunities, allies, and discourses [31, 32]. Movement actors can themselves create political opportunities [33], but varying political systems are more or less receptive to protest and political change [32]. According to political opportunity theory, social movement action is possible when political agendas and institutional structures have some openness to the claims of social movement actors [33, 34]. The promotion of user involvement in policy documents in Sweden could thus be analyzed as creating a receptiveness to change initiatives from the user movement in the institutional environment.

In the Nordic countries, social movement actors have often chosen less confrontational strategies, and rather focused on collaboration with authorities [35]. In these countries, boundaries between social movement actors and administrators or service providers are to some extent blurred, through an expectation of shared interests (cf. [36]). This pattern is interesting in relation to the assumptions of political opportunity theory, where favorable political contexts are assumed to lead to more active protests [33, 34]. To some extent, the Nordic context diverges from these assumptions, where favorable institutional contexts have led to extensive social movement activity, but seldom in the form of radical protest [31]. UFM reflects these patterns, as social movement activities are focused on collaboration, where the user movement through the practice assumes a consultative role in the development of welfare services. The corporative tradition of these countries [36] means that cooperation rather than conflict is likely to be associated with an increase in political opportunity.

Negotiations with decision-makers are central in user collectives’ ability to seize political opportunity. In the mental health sector, there are different understandings of how services are to be organized among different professional and lay groups. Such concerns are continuously negotiated and subordinate actors can affect bargaining, for instance, through different kinds of protests [37]. The process and outcome of negotiations are connected to the type of resources that groups have access to, which are further linked to different movement strategies [38]. Except for the institutional context, the organizational factors that are important in forming tactics are the size, degree of collaboration, professionalization, and formalization of social movement organizations. Increased size and resources amount to increased capacity [39]. Ties between protest groups and authorities also affect the negotiation strategy, where higher levels of trust mean that a search for mutually beneficial solutions is more likely [38]. Professionalization of social movement organization through specific training can further increase competency in advocacy-related skills, but it can also decrease the focus on more radical social change. Finally, the level of formalization of organizations and the specialization of tasks affect strategy and activities by being related to both a growth in size and an increased ability to secure government funding [39]. In our discussion on negotiations in the UFM practice, we specifically address how collaboration, formalization, and professionalization affect user organizations’ bargaining stances.

## Methods

This study is focused on synthesizing general patterns in 136 Swedish UFM reports in order to characterize the phenomenon. A project group was formed during the planning phase of this study and its overarching research project. Besides the researchers, the project group has around ten members that represent the user movement in different geographic locations. Representatives of municipality-based mental health services with experience as commissioners of UFM were also included in the project group. Through reoccurring meetings, the project group provided input throughout the research process by contributing to formulating core concerns and to the analysis. The benefits of involving people with lived experience in research has been recurrently discussed (e.g. [40, 41]) and the involvement of these stakeholders in the research process is also important to ensure the translation of research results to practice.

### Data

We have mapped UFM reports conducted since 2015 in Sweden, compiled in a database by the NSPH (The National Partnership for Mental Health, the umbrella organization of Swedish mental health user organizations). This database contains 122 reports and constitutes a unique source of data that enables an analysis of the features of and patterns in Swedish UFM reports. The database further covers the vast majority of UFM conducted in the mental health sector. We gained access to the reports in this database through contact with NSPH. In addition to this database, we have added 14 UFM reports conducted by the user organization Verdandi, through contact with the organization. Verdandi mainly focuses on addiction, but we have included reports attending to service sites that are focused on mental health concerns. In total, 136 reports were included in the study. Regarding ethical considerations, informed consent has been obtained from the organizations from which the reports were collected. Furthermore, this study is focused on mapping patterns in UFM reports that are publicly available (frequently by being posted by the user organizations at their websites). The reports further cover data that already has been anonymized. Consequently, this study does not handle any sensitive data that can be tracked to unique individuals.

### Analysis

Initially, a selection of 13 UFM reports were sampled for a preliminary analysis. The selection was based on the ambition to reflect the diversity among the reports regarding geographic location (south-, mid-, and north of Sweden), executant organization (including both large but also smaller user organizations), commissioner (region- and municipal commissioner), and the type of service examined (accommodation services, vocational rehabilitation services, in- and outpatient care). During this pilot study, the individual reports were read and analyzed with the aim of developing a data matrix that would be applied to analyze all UFM reports. We singled out a number of background characteristics such as the year performed, municipality/geographic context, possible revisit, executant organization, commissioner, the principal organization of the service site, and the type of service examined. We also decided to include a number of method-related variables, describing the number of user monitors, number of informants, the report length in words, the methodological approach, and external method support. The data matrix was developed in collaboration with the project group. We thereafter scanned all reports for the information covered by the data matrix. All information was coded into SPSS and descriptive statistics and cross tables were employed to analyze the material. This initial analysis was performed to map the reports according to general characteristics, and thereby provides quantitative information of UFM in relation to where, how and by whom the method is conducted.

At a later stage we performed a directed content analysis [42] of a selection of 20 reports. Our ambition was to include a variety of UFM reports that covered different service contexts, methodological approaches, geographic contexts, and executant organizations. We strived to include reports that were representative of the larger sample but also to include reports that showcased the variety in UFM with regards to the methodological approach and service context. Through our pilot study, we had identified four dimensions as central parts in the UFM reports: *the commission, the method, the results, and the development proposals*. These four dimensions were applied as ‘predetermined categories’ ([42], p. 1281), developed through our initial reading of the reports. Drawing inspiration from the structured analytical approach of directed content analysis [42], we separately coded these different sections of the reports. Codes and associated text segments were sorted to analyze similarities and differences in relation to these dimensions among the included reports. This qualitative analysis was applied to provide a descriptive overview of the UFM reports with additional information on how UFM is conducted, what the objectives of UFM are, but also insights into patterns in results and development proposals presented in the reports. The sampling of reports and the focus of the analysis were elaborated on in the research group and also in the larger project group. Analytical memos were written by the researchers, based on discussions in the project group. The project group primarily contributed to the analysis by providing background information on empirical findings. In the following, we will discuss general patterns in Swedish UFM reports, based on our initial mapping analysis and the following qualitative analysis. All numerical distributions are based on our initial mapping analysis of 136 reports.

## Results

Our initial mapping analysis of 136 UFM reports shows that around the same number of reports were performed annually during the time interval 2015–2019 (due to Covid-19 restrictions, the number of reports from 2020 to 2021 is limited), see Table 1. 29.4% of the reports were followed-up with a revisit to the service site and the number of follow-ups has further increased over the years. From 2015 to 2017, 16.9% of the reports were followed-up, compared to 43.1% of the reports from 2018 to 2021. This pattern could be seen as a methodological development, but also as a reflection of advanced positions on the part of the user movement, since it means that user monitors not only examine a service site, but also return to evaluate what changes has been made. This means that services are, to a higher extent, held accountable for what actual change has occurred as a result of user involvement initiatives.

**Table 1:** Number of UFM reports according to year

Year	Frequency	Percent
2015	21	15.4
2016	26	19.1
2017	24	17.6
2018	24	17.6
2019	33	24.3
2020	7	5.1
2021	1	0.7
Total	136	100.0

### Who Carries Out UFM—and Where is it Performed?

Our mapping analysis illustrates that a couple of executant organizations are dominant in the field. Most UFM in the mental health sector is conducted by organizations that are based in metropolitan areas in the middle and south of Sweden. A more limited number of UFMs are conducted by smaller organizations in less populated areas. This pattern relates to the resources available for the implementation of these types of systematic methods for user involvement in different contexts. It raises questions regarding the conditions for such systematized methods in areas with a smaller population base or a less developed self-organization of service users.

Most UFM is conducted by local user organizations, but through a national UFM project, a “mobile team” of user monitors has also performed UFM elsewhere in the country. There are further instances where a research and development unit constitutes the overriding organization for the UFM. For a couple of UFM projects, municipality-based organizations have functioned as the organizer. According to the project group, this has been protested by the user movement. Our data reveals that most executant organizations are today connected to the user movement, thereby offering greater autonomy in relation to the service system.

The mapping analysis further reveals that most of the UFM reports cover municipality-based services (62.5%) but region-based services are also represented (25.7%), see Table 2. The dominance of municipality-based services has, however, decreased over time, indicating a pattern of increased interest in this method among region-based mental health services. The low frequency of privately run services examined through UFM can be related to the limited involvement of such actors in the Swedish mental health sector [25]. Furthermore, patterns of less established communication channels between private providers and the user movement have been discussed in our project group.

**Table 2:** Principle organization of the service examined

Principal organization	Frequency	Percent
Municipality	85	62.5
Region	35	25.7
Other	13	9.6
Private	3	2.2
Total	136	100.0

Almost half (44.9%) of the UFMs were conducted on accommodation services, see Table 3. Outpatient care (14.7%) and day centers/vocational rehabilitation (13.2%) are the second and third most common service type in the sample. However, the analysis shows that the dominance of accommodation services has decreased over the years.

**Table 3:** Type of service examined

Service type	Frequency	Percent
Accommodation services	61	44.9
Outpatient care	20	14.7
Day centers/vocational rehabilitation	18	13.2
Inpatient and outpatient care	15	11.0
Inter-organizational service	12	8.8
Inpatient care	8	5.9
Other	2	1.5
Total	136	100.0

### Who Makes the Commission—and How is it Formulated?

In the mapping analysis of 136 reports, we found that the commission of a UFM was in 76.5% of the cases derived from higher administrative levels. In 21.3% of the cases, the commission came directly from the service site in question (three reports did not specify the commissioner). This could reflect a tendency of top-down approaches to user involvement–where knowledge of the method is more prevalent among managers and officials at higher administrative levels. However, this pattern mainly reflects the fact that the user organizations that carry out the most UFM have ongoing contracts made with higher administrative levels to deliver a certain number of reports annually. These long-term contracts are thus important for securing continuity in implementation.

The UFM reports include a section that describes the commission and the aim of the UFM. The qualitative analysis demonstrates that many describe as the aim to examine service users’ experiences as part of development work, or to evaluate services from a service user perspective in relation to what is good, not so good, and how services can be developed. UFM is further described as contributing to securing the quality of services from a service user perspective. UFM thus often aims to provide both a current description of how services are perceived by service users and also a development perspective. The restricted focus of most UFM to a specific service site could risk a failure to adopt a comprehensive view of service users’ lives and concerns by turning the focus to issues of “consumer satisfaction”. However, some UFM reports, and mainly those that are not restricted to a specific service site, focus on broader issues. Such reports can, for instance, have the aim of exploring how user participation can be developed in a certain region:

The aim of the UFM is to examine [the region and municipality’s] work with the state subsidy for mental health from a service user and participation perspective. The aim is also to develop a basis for … future work with user participation within the realms of this investment and in the general work for strengthening user participation …

Another variation among the UFM reports concerns whether representatives of the service site examined should contribute to formulating the focus of the UFM. Some reports describe how the commissioner has requested certain areas that the UFM should encompass. Others describe it as a central tenet of UFM that representatives of the service site do not inform its focus:

The user monitors have [developed] an … interview guide as a frame for the interview work. This could be said to be one of the foundational bolts of the UFM; that it is the user monitors and not the services who determines what questions are to be posed.

In addition to the specific aim of the UFM, the reports frequently include a description of UFM as a general approach, to discuss its central assumptions and benefits:

People with their own experience … have extensive knowledge … and they have a unique inside perspective. When this knowledge resource is utilized, it can contribute to … developments of the service … In UFM, both the service users' and the user monitors' experience of mental ill health is … highlighted as an asset. The service users have knowledge of current activities and the user monitors can, through their own experience, design relevant interview questions, interview the service users in an equal encounter and ask good follow-up questions.

How UFM is based on the service user informants’ and the user monitors’ experiential knowledge is especially highlighted, reflecting a focus on UFM as a method of integrating experiential knowledge in the knowledge base of mental health services.

### How is UFM Conducted?

A number of UFM models have been developed in Sweden that represent somewhat different approaches. Some models have been developed by actors representing the service system—and as a response to this, user organizations describe their models as “user governed, user focused monitoring”, to underscore their greater autonomy and user ownership. Furthermore, different models vary in their methodological approach. With regards to the methods employed, our initial mapping analysis established that qualitative and interview-based methods (80.9%) are dominant in the sample. 11.8% employed quantitative methods and 5.1% used a mixed-method approach. Three “meta-reports” were also included, that had compiled results from multiple reports. 27.2% of the reports describe some kind of external method support from, for instance, a research and development unit. A vast majority, 88.2% of the reports, only include service users as informants, but a couple of the reports also include staff, relatives, or officials. The number of informants vary from 3 to 791 (median = 10) and the number of user monitors vary from 2 to 13 (median = 5). A typical report is around 8000 words long, but the reports vary from 1400 to 50,000 words.

The qualitative analysis shows that many reports describe utilizing a UFM coordinator, who assumes the main responsibility for practical arrangements associated with the UFM. The user monitors have received training in the method, most often by the executant organization. In some models, user monitors that have experiences that match those of the service users at the service site in question are selected for the UFM. Different procedures have guided the design of the interview guides or surveys. Some interview guides have been worked out by the user monitors, some have involved deliberations with representatives of the service site and some have, as mentioned above, involved input from a research and development unit.

The process of conducting UFM involves several steps. Initially, the user monitors or the UFM coordinators meet with representatives of the service site, which is followed by a visit to a workplace meeting where the staff is informed of the upcoming UFM. A couple of different recruitment strategies are reported. In some cases, staff is given the assignment of informing service users of the possibility of participating in UFM. In other cases, the user monitors hold an inspirational meeting with the service users. In the case of interviews, the user monitors often offer both individual and group interviews, but individual interviews are the most common form. Surveys can either be conducted in person, through letter surveys, or web links.

The reports include limited information on the process of analyzing the data. Some describe all user monitors being involved in this step, whereas other reports describe how the UFM coordinator plays a central role in analyzing the data and writing up the results. The reports are finally delivered to management and staff at the service site. From our project group, we have learned that another divergence regards ownership of the UFM reports. Some organizations co-own the reports and are therefore able to use it for their own purposes and distribute it to a public audience. This has, however, been a contested issue and some organizations do not have such ownership of the UFM reports that they produce.

### How are Results Presented and What Areas do They Cover?

Patterns in results and in development proposals are derived from our qualitative analysis. The results section of the reports often starts with a presentation of the demographic of the informants. Some reports further present a summarized assessment of the results, in relation to the aspects of care and support that the service user informants are pleased with, as well as the aspects that have room for improvement. Most reports employ qualitative methods and report quotes from the informants. In some qualitative UFM models, the presentation of the results does, however, have quantitative features by including frequencies:

A3. Do you miss any form of daily leisure activities?

Number of respondents: 21

Answer: Eleven respondents answer yes, ten respondents answer no.

Quote: “Yes, during the weekends, the day centers are always closed during the weekends.”

The exact number of informants who have chosen a specific answer is reported in this model. Such a presentation of the results closely aligns with the informants’ answers, and could thus be analyzed as mainly capturing the opinions and knowledge of the service users at the service site. Other UFM models more explicitly include the voices of the user monitors in the presentation of the results. Some models, for instance, add a section called “the user monitors’ reflections” after each theme, where the user monitors analyze the results:

We as user monitors get the impression that there are not a lot of activities … We monitors have the experience of psychiatric accommodation, where there is a risk of the residents getting “stuck” at home and not getting out much. Hiring a staff member who is responsible for activities is a way of counteracting such a development.

In these models, the user monitors’ own knowledge and preunderstanding are made explicit. In other models, the informants’ answers have undergone further qualitative analysis, where inductively derived themes are presented and discussed in relation to theoretical concepts. UFM reports that are more analytical in nature often examine efforts that are less tied to a specific service context, such as user councils or general work with user participation, or a state subsidy, within a region.

The main focus of many UFM reports rests on service users’ satisfaction with their care and support, by providing a snapshot of how services are experienced. Less emphasis is placed on whether the service user informants conceive that they have benefitted from the service programs. However, some reports include a question that focuses on the significance of the care or support over time: *“Has the treatment affected your life situation—if so, in what way?”* The reports showcase some variation with regard to the areas that they cover, but there are also themes that are reoccurring. The Swedish mental health system is, to a high extent, sectorized [43], with a multitude of service actors and government agencies commonly involved in the individual’s support. This is reflected in an emphasis on themes that relate to inter-organizational *cooperation* and *continuity*. An area that further relates to questions regarding *care planning and information*:

One respondent was very dissatisfied with the planning, based on having seen the beginning but not the end of the planning … This created … uncertainty about where to turn after discharge as no outpatient contact had been established during the treatment time at the ward.

The reports often include some themes that are more focused on practical concerns. Many reports cover *activities*, for instance asking whether the service user informants are content with the activities that are offered. Another common area is *accessibility*, for instance exploring different groups of service users’ ability to travel to and to access the service site. Furthermore, experiences relating to the *premises and the food* offered are often examined.

Another common area concerns *participation and influence*, both with regard to the individuals’ own care planning but also in relation to service users’ influence over decisions at an organizational level. In terms of a recovery perspective, some reports have specific areas that relate to *inclusion and empowerment*. Common themes further cover relationships with staff, for instance *personal treatment and competency*. This category of results both covers how the service user informants perceive treatment from staff members, and also whether they understand staff to have sufficient knowledge of, for instance, treatment options or diagnoses. *Problems and conflicts* with staff are commonly reported through a specific theme in the reports.

### What Themes do Development Proposals Concern?

The reports frequently present development proposals in relation to the different themes covered in the results section. The development proposals can be quite concrete, especially proposals that concern the premises, food, and activities. These proposals can be directly drawn from propositions made by the service user informants during the interviews. Development proposals frequently concern resources, both in relation to finances and staff characteristics. A common development proposal is, for instance, to hire a specific staff member to deal with a problem identified, such as a lack of activities or a lack of structure for user influence. Some proposals also reflect a focus on a recovery perspective, by placing emphasis on the hopes and preferences of the service user:

The implementation plan: Inform what an implementation plan is and make an implementation plan together with the resident. It is important that dreams, goals, interests, desires, and strengths are included. We also propose that each resident should have at least one social support time per week, where the resident decides the activity as a way to increase self-determination.

The content of development proposals varies in relation to the specific service site, but also in relation to the type of service examined. Questions that regard personal treatment are, however, seldom one of the major issues. Often the user monitors instead compliment the staff on personal treatment, encouraging them to keep up the good work. In general, major concerns instead relate to problems regarding continuity in care, inter-organizational cooperation, information, and influence. Many UFM reports highlight a lack of information regarding treatment options and rights, and how this undermines the individual’s influence over care planning. Sharing information with service users has been described as the first step towards participation [44], and the user monitors recurrently identify this area as important in order to move towards higher levels of participation and shared decision making.

## Discussion

The struggle of user movements for participation has been a driving force for initiatives related to user involvement and UFM both internationally [4] and in Sweden. However, the importance of involving user groups in service and policy development has also been adopted by public sector actors. UFM can thus be analyzed in relation to both top-down and bottom-up approaches, originating in grassroots initiatives, but which are today also promoted by public authorities (cf. [13]). Drawing on our results, we will in the following section discuss negotiations and political opportunities in the UFM practice.

### Political Opportunities in the Institutional Context of UFM

Social movement actors’ strategies and successes are related to the political opportunities that are available in the institutional context. When institutional conditions are favorable, an increase of social movement activity and protest is likely to occur [32]. In Sweden today, user involvement is emphasized as a priority issue in government commissions of inquiry [7, 8]. There has, for many years, been an increased awareness of the value of integrating user collectives’ voices into service system developments. The establishment of user involvement initiatives such as UFM in the Swedish context can be connected to such policy demands, which create political opportunities for the user movement. However, it is important to note that rather than a focus on radical protest, these movement actors are increasingly engaged in systematic methods such as UFM, that involve some form of collaboration with service providers (cf. [28, 45]). Aaslund [31] similarly notes that fertile political conditions in the Nordic countries have led to an increased focus on collaboration rather than increased protest among social movement actors.

An aspect of political opportunities in the institutional environment, especially important to the UFM practice, are the long-term contracts to perform UFM that have been established with public sector actors. Our results reveal that the organizations that perform most UFM have such general contracts with commissioners at municipal or regional levels. Our project group has informed us of how the establishment of long-term contracts has been proceeded by negotiations, where the user movement through deliberations with public sector actors has entered into such agreements. These contracts form an important condition for creating continuity in the implementation of UFM. Such contracts further enable long-term planning that facilitate a better work environment for user monitors. These insights are relevant to a general discussion on how to develop sustainable user involvement. It serves as an illustration of the importance of securing long-term financing and continuity in demand in order to create durability in user organizations’ role as a counterpart to public authorities. However, it also underscores the importance of actively fostering strategies to protect user organizations from being co-opted as a result of such close collaborations (cf. [28, 46, 47]).

### Negotiations in the UFM Practice

Negotiations that concern autonomy and user control are visible throughout the process of conducting UFM. One such point of negotiation concerns who is to carry out UFM, where there are examples of municipalities that have assumed the role of UFM organizer. In such instances, the degree of user control in UFM has been a contested issue. The present analysis illustrates that deliberations further regard who is to formulate the aim and the focus of the UFM, where user autonomy and control are described as a central tenet of the method in the reports included in this study. Several authors have discussed challenges presented to autonomy, by user organizations engaging in multiple roles and in close collaborations with authorities (e.g. [48–50]). However, Pestoff et al. ([51], p. 594) discuss how ‘considerable autonomy’ can be available if strategic opportunities are utilized. Negotiating concrete structures in the UFM practice to protect autonomy and user control, is a strategy employed by the user movement to make the most of the opportunities for influence available.

UFM can be analyzed as a practice that moves between the individual, organizational, and structural level. The method examines the experiences of the individual service user and can be used as a resource for user movements to aggregate structural information based on local conditions. The current analysis shows that the main focus of UFM rests on organizational development. However, in order to protect the user movement’s autonomy in UFM, it is important to resist a primary role as a public sector consultant in the development of a service site. The value of UFM for the user movement’s own purposes needs to be protected, for instance through the method being used to build a collective knowledge base, and also to gain system-related knowledge that is valuable in advocacy work. The risks of consultative involvement ([52], p. 497) further motivates UFM exploring issues relevant to service users’ lives, unrestricted by the service site as an analytical frame. Current examples of meta-studies, where service users’ experiences from a range of services are compiled by the user organizations, could be one way to generate such collective knowledge and to adopt a broad focus on exploring central concerns to service users.

Through UFM, the experiential knowledge of service users in local service contexts is compiled. UFM is further based on the experiential knowledge of the user monitors, who design the UFM, gather information, and analyze the results. The method can thus be connected to a current development towards increased legitimacy and inclusion of experiential knowledge in mental health service systems (cf. [14, 53–55]). The reports included vary in relation to whose knowledge is at the core, where some UFM models emphasize the informants’ voices while others more explicitly include the voices of the user monitors. A trend towards demands of objectivity in the user monitors’ role has been described in our project group. This may be an indication of the practice being influenced by institutional norms of a formal evaluation being objective. Similarly, Meriluoto [56] has discussed the pressures placed on so called experts-by-experience to adapt a neutral position. Yet another negotiated issue concerns the ownership of UFM, where some organizations require reports to be co-owned in order to conduct a UFM. For other organizations, this is still a matter of negotiation. The current analysis revealed a development towards UFM more frequently being followed-up. This contributes to increasing demands of accountability on the part of service providers, in relation to the actual change that has taken place. Based on prevalent discussions of tokenism that surround user involvement practices (e.g. [57, 58]), demanding accountability on the part of authorities is a current challenge to user movements [50]. Increased accountability can also be effective against so called ‘consultation fatigue’ [52] by being used to track the actual change achieved through user involvement initiatives. It can thereby be used as tool for the user movement to form their strategies regarding time and resource investment.

### Professionalization and Trust

UFM in itself demands a certain degree of professionalization and formalization of user organizations in order for the method to be systematically conducted and to assure continuity. Our analysis illustrates how user organizations through UFM assume the role of educator and employer of user monitors, and the formal responsibilities that such roles demand. The stance in UFM negotiation processes is further affected by the professionalization and formalization of user organizations, as these organizational factors are associated with an increase of resources and weight in bargaining processes [39]. Furthermore, professionalization and formalization relate to issues of trust between user organizations and public sector actors. When user organizations adopt systematic methods that are associated with increased formalization and professionalization, such as UFM, they are likely to become more understandable to their public counterparts. Rather than engaging in sporadic protests, methods such as UFM are more closely aligned with the logic of the public sector, which builds trust. This pattern can be related to isomorphism, where organizations gain legitimacy by adapting to institutional environments [59]. When public sector actors secure long-term contracts for UFM that provide user organizations better conditions through stable funding, this trust is further mutually built. As mentioned, higher levels of trust frequently lead to a search for solutions that are mutually beneficial [38]. However, this development is also associated with risks of professionalization and a potential stratification of user groups in involvement practices discussed in prior research, where only service users with specific resources, positions and training are represented (e.g. [60–62]). Moreover, this poses questions concerning what is required of user organizations in order to be seen as professional and trustworthy in the eyes of their public counterparts. Further research will need to explore if these frames of professionalism limit the focus of UFM.

## Conclusion

Arguments for increased user involvement can be broken down into three categories: those related to developing democratic participation, those related to power redistribution, and those related to service system adjustments [63]. Involving service users in service development has been discussed in relation to more ethical systems from a democratic and human rights perspective and arguments that focus on the quality development of services conclude that more efficient services can be created [3, 63]. UFM can thereby contribute to refocus the quality development of services to key concerns from a service user perspective, and to strengthen organizational readiness to integrate service user voice and knowledge. For the user movement, UFM further has the potential to contribute to a broader knowledge base, through increased knowledge of service and support system impediments and grassroots experiences of these. Such knowledge is key to inform the focus of the movement, in advocacy, as well as other, endeavors.

In this study, we have synthesized patterns in Swedish UFM reports to characterize UFM as a method for user involvement. Drawing on these results, we have discussed how user groups take advantage of political opportunities in the institutional context by engaging in negotiations with service providers. Long-term contracts between user organizations and service providers are important in order to create a sustainable implementation of UFM. However, strategies to protect user autonomy must be carefully considered and employed in relation to such long-term collaborations. Concerns regarding a narrow focus in UFM on consumer satisfaction have been highlighted. Future studies will need to examine the conditions that need to be present in order to engage in these kinds of systematized methods of user involvement. Moreover, specific strategies to resist co-optation need to be explored.
